# FEASIBILITY OF RECRUITING PEOPLE WITH MILD COGNITIVE IMPAIRMENT IN THE CONTEXT OF HEART FAILURE

**DOI:** 10.1093/geroni/igae098.3658

**Published:** 2024-12-31

**Authors:** Miyeon Jung, Susan Pressler, Dustin Hammers, Liana Apostolova

**Affiliations:** Indiana University School of Nursing, Indianapolis, Indiana, United States; Indiana University, Indianapolis, Indiana, United States; Indiana University School of Medicine, Indianapolis, Indiana, United States; Indiana University School of Medicine, Indianapolis, Indiana, United States

## Abstract

Recruiting people with mild cognitive impairment (MCI) with another chronic condition such as heart failure (HF) can be arduous. Our investigative group will discuss the challenges encountered while recruiting older adults with both MCI and HF using data from a pilot study testing the efficacy of cognitive interventions to improve cognitive function and the strategies to overcome them. Initially, eligibility criteria included age ≥65 years, HF confirmed by echocardiography, and MCI defined using a 2-step process: (1) Montreal Cognitive Assessment (MoCA) ≤23; and (2) diagnostic consensus of MCI based on the presence of cognitive impairment in the absence of functional decline. Enrollment began on 4/3/2023 by screening Cardiology and Neurology clinics patients. Only 12 participants were enrolled over the next 7 months (rate=1.5 participants/month) due to high screen failure rates (59%) owing to MoCA performances above the eligibility threshold and low recruitment rate (5%). To meet recruitment goals (8 participants/month), eligibility criteria were modified by lowering the age cutoff from 65 to 55 years and removing the MoCA screen and the MCI requirements, while adding the requirement of subjective cognitive concern allowing both those with normal cognition and MCI but not dementia. Phone recruitment was added by screening electronic health records of people who diagnosed with HF. 7 months after implementing the modifications, additional 58 participants were consented exceeding our recruitment goals (69% of those consented=MCI, 26%=normal cognition, 5%=dementia/excluded from the study). In conclusion, feasibility of our original strategies recruiting older adults with both MCI and HF was not supported.

